# Microheterogeneity-induced conduction slowing and wavefront collisions govern macroscopic conduction behavior: A computational and experimental study

**DOI:** 10.1371/journal.pcbi.1006276

**Published:** 2018-07-16

**Authors:** Tanmay A. Gokhale, Huda Asfour, Shravan Verma, Nenad Bursac, Craig S. Henriquez

**Affiliations:** Department of Biomedical Engineering, Duke University, Durham, North Carolina, United States of America; University of California San Diego, UNITED STATES

## Abstract

The incidence of cardiac arrhythmias is known to be associated with tissue heterogeneities including fibrosis. However, the impact of microscopic structural heterogeneities on conduction in excitable tissues remains poorly understood. In this study, we investigated how acellular microheterogeneities affect macroscopic conduction under conditions of normal and reduced excitability by utilizing a novel platform of paired *in vitro* and *in silico* studies to examine the mechanisms of conduction. Regular patterns of nonconductive micro-obstacles were created in confluent monolayers of the previously described engineered-excitable Ex293 cell line. Increasing the relative ratio of obstacle size to intra-obstacle strand width resulted in significant conduction slowing up to 23.6% and a significant increase in wavefront curvature anisotropy, a measure of spatial variation in wavefront shape. Changes in bulk electrical conductivity and in path tortuosity were insufficient to explain these observed macroscopic changes. Rather, microscale behaviors including local conduction slowing due to microscale branching, and conduction acceleration due to wavefront merging were shown to contribute to macroscopic phenomena. Conditions of reduced excitability led to further conduction slowing and a reversal of wavefront curvature anisotropy due to spatially non-uniform effects on microscopic slowing and acceleration. This unique experimental and computation platform provided critical mechanistic insights in the impact of microscopic heterogeneities on macroscopic conduction, pertinent to settings of fibrotic heart disease.

## Author summary

It is well known that perturbations in the heart structure are associated with the initiation and maintenance of clinically significant cardiac arrhythmia. While previous studies have examined how single structural perturbations affect local electrical conduction, our understanding of how numerous microscopic heterogeneities act in aggregate to alter macroscopic electrical behavior is limited. In this study, we utilized simplified engineered excitable cells that contain the minimal machinery of excitability and can be directly computationally modeled. By pairing experimental and computational studies, we showed that the microscopic branching and collisions of electrical waves slow and speed conduction, respectively, resulting in macroscopic changes in the speed and pattern of electrical activation. These microscale behaviors are significantly altered under reduced excitability, resulting in exaggerated collision effects. Overall, this study helps improve our understanding of how microscopic structural heterogeneities in excitable tissue lead to abnormal action potential propagation, conducive to arrhythmias.

## Introduction

Both experimental [1] and clinical [2] studies have shown that the presence of non-conducting tissue heterogeneities in the myocardium can facilitate arrhythmia induction and maintenance. These tissue heterogeneities vary greatly in size, shape and distribution. Arrhythmogenic roles of macroscopic heterogeneities in cardiac tissue, such as the ostia of the pulmonary veins, the heart valves, and infarct scar, have been extensively studied [3]. Mines first described anatomic reentry in cardiac tissue [4] in 1914, and more recent work in animal and tissue culture models has examined the role of macroscopic heterogeneities as attachments points for anatomical reentry [5–7]. In contrast, the role of microscopic heterogeneities, less than 1 mm in size, such as localized fibrosis and non-conductive cell populations, is incompletely understood due to the lack of experimental and computational tools to investigate electrical conduction simultaneously across spatial scales.

The importance of cardiac microstructure on propagation and arrhythmogenesis was first described by Spach and colleagues [8–11], who showed that while conduction appears continuous on the macroscopic level, it is inherently discontinuous on a microscopic level. The advent of microfabrication techniques for creating patterned extracellular-matrix protein surfaces or hydrogels has enabled generation of complex 2D [12–14] and 3D [15] cell cultures that allow systematic *in vitro* studies of cardiac structure-function relationships. These studies have revealed that isolated tissue expansions and contractions can cause conduction slowing [16–18] and conduction speeding [19], respectively, while tissue geometries with several “branches” in series exert “push” and “pull” effect on microscopic conduction [20]. Although some experimental evidence exists that multiple heterogeneities in aggregate can lead to conduction block [21] or reentry [22], a mechanistic understanding of how regions of microheterogeneity affect conduction has remained elusive due to the inability to simultaneously examine conduction on the microscopic (μm) and macroscopic (cm) scale.

One advantage of computational studies over experimental studies is the ability to examine conduction at a variety of spatial and temporal scales. Several groups have used *in silico* models to study the effects of microfibrosis on both macroscopic [23–26] and microscopic [18,27] conduction. Ten Tusscher et al. showed that diffuse fibrosis slows macroscopic conduction, increases vulnerability to spiral wave formation, and slows spiral wave rotation [23]. However, the large spatial discretization of their tissue model did not permit a mechanistic understanding of the effects of fibrosis at microscopic scale. In addition, most simulation studies have been conducted without a direct comparison with matched experimental studies.

In this work, we developed a combined experimental and computational framework to study how defined acellular microheterogeneities of varying size affect macroscopic behavior under normal and low excitability conditions. By utilizing micropatterning techniques [14] and a previously developed HEK293 excitable cell line (“Ex293” [28]), we generated engineered tissue networks with microscopic square holes of varying size and spacing and applied electrical pacing, pharmacological agents, and optical mapping to assess the impact on macroscopic action potential propagation. These tissue structures were reconstructed *in silico* using a previously validated computational model of the Ex293 cell line [29] to reveal mechanisms underlying the observed experimental results. Our findings build on the previous work in isolated tissue expansions and demonstrate that the microscopic slowing and speeding due to source-load mismatches near and around obstacles do not cancel at the macro-scale, but rather directly affect the wavefront shape, velocity and safety of conduction under both normal and reduced excitability conditions.

## Results

### Conduction in microheterogenous monolayers

**Fig 1:**
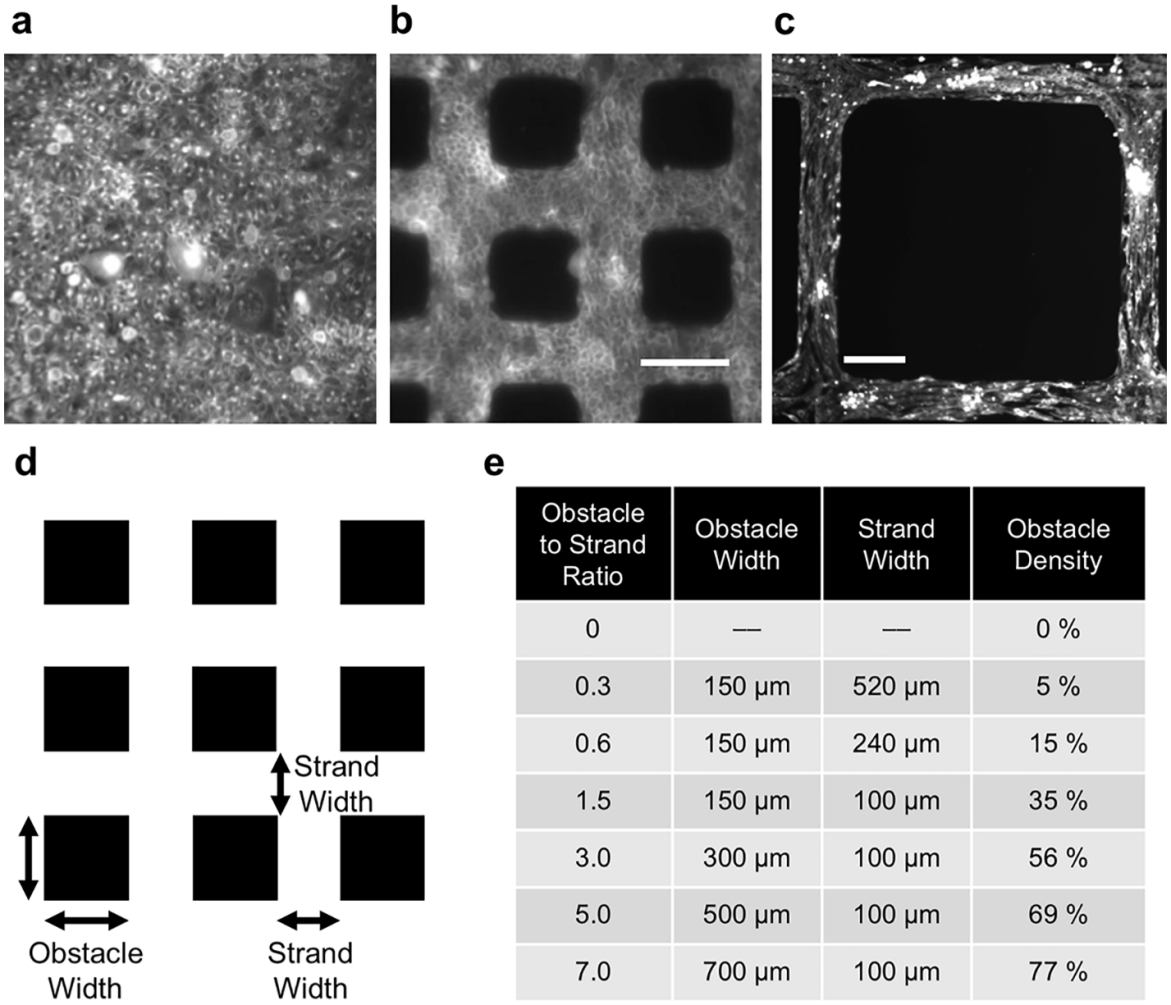
Micropatterning via photolithography resulted in cultured monolayers of Ex293 cells around acellular regions that varied in size and spacing. (a) Homogenous monolayer; obstacle-to-strand ratio: 0; (b) 150 μm x 150 μm obstacles, separated by 100 μm strands; obstacle-to-strand ratio: 1.5; (c) 700 μm x 700 μm obstacles, separated by 100 μm strands; obstacle-to-strand ratio: 7.0 (scale bar = 150 μm); (d) Schematic depiction of tissue obstacle structure; (e) Obstacle and strand widths, and obstacle percent density, for each degree of heterogeneity.

Culturing Ex293 cells for 4 days on micropatterned coverslips yielded confluent monolayers that contained strands and acellular square regions (obstacles) with various sizes and separation (Fig 1). At lower obstacle-to-strand ratios, Ex293 cells were round and isotropically arranged, while in monolayers with large acellular regions, we observed some degree of cell alignment along the axis of intra-obstacle strands (Fig 1a–1c).

**Fig 2:**
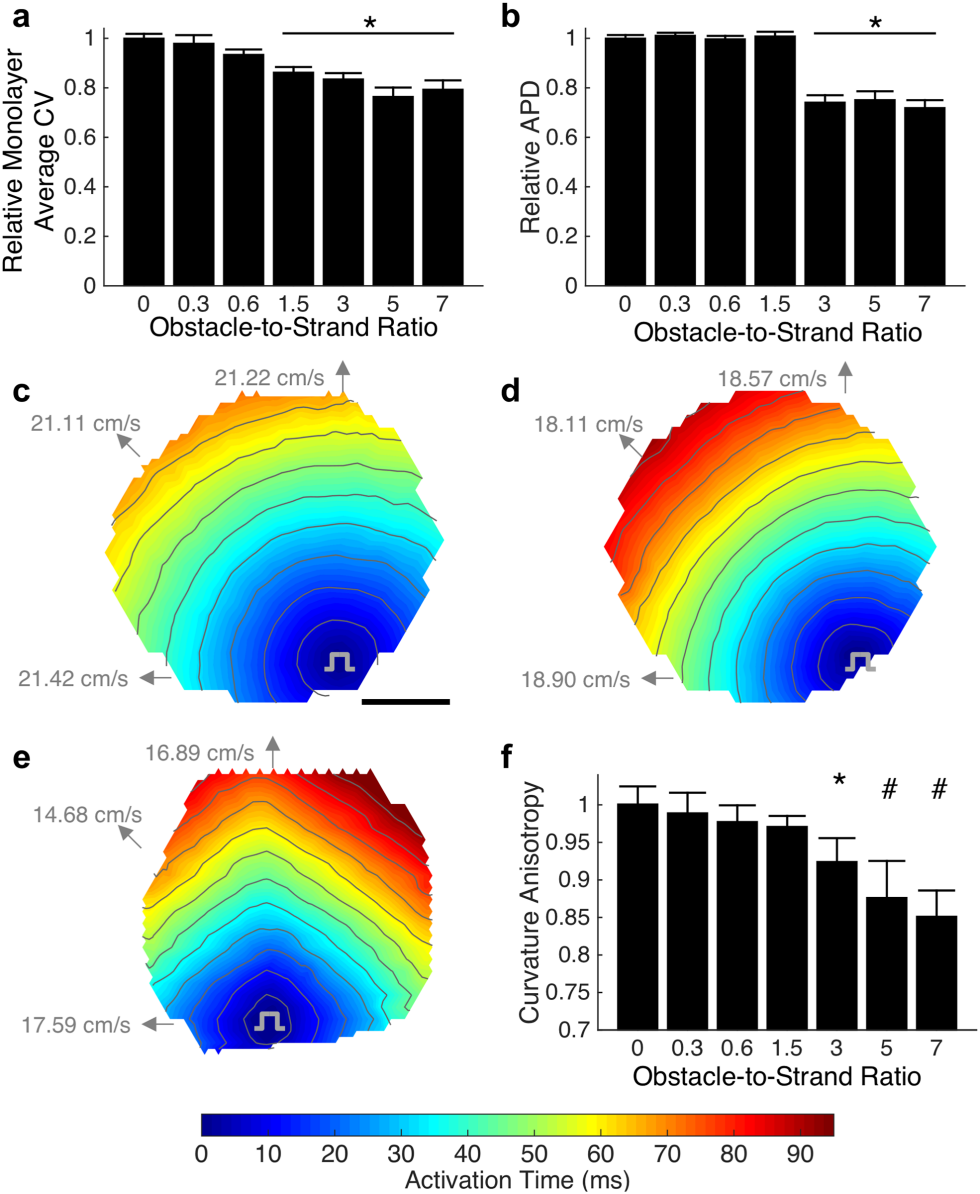
Effects of microscopic heterogeneity on macroscopic conduction. (a) Increasing degree of heterogeneity, as characterized by the obstacle width to strand width ratio, leads to slowing of macroscopic conduction (mean ± se; n = 13–68 monolayers; F(6,217) = 11.53. p < 0.0001; Asterisk indicates significant difference from homogenous case, p < 0.05; values represent average CV across each individual monolayer). (b). Shortening of action potential duration is observed at high obstacle-to-strand ratios (mean ± se; F(6, 217) = 42.7. p < 0.001). (c-e) Macroscopic activation maps becomes increasingly anisotropic as obstacle-to-strand ratio increases from 0 (c) to 1.5 (d) to 7.0 (e). Directional conduction velocities are as indicated. Average conduction velocities across these representative monolayers are 21.28 cm/s (c), 18.56 cm/s (d) and 16.79 cm/s (e). Scale bar = 5 mm; Activation isochrone lines at 8 ms spacing. (f) Quantified anisotropy of 1 indicates isotropic conduction while √2/2 indicates a diamond shape isochrone (mean ± sd; n = 10–15 monolayers; F(6,72) = 42.5. p < 0.001; * and # indicate significant difference from all lower obstacle-to-strand ratios, p < 0.05).

Monolayers were stimulated by a point electrode and conduction velocity (CV), derived from optical mapping, was found to inversely correlate with obstacle-to-strand ratio (Fig 2a). In particular, pronounced CV slowing was observed as the obstacle-to-strand ratio increased from 0 to 1.5 (9.7% per unit ratio), followed by a modest CV slowing for larger ratios (1.4% per unit ratio) where the mean action potential duration (APD) was also decreased (Fig 2b). The largest CV decrease of 23.6%, from a control CV of 19.2 ± 0.5 cm/s (mean ± se), was observed at an obstacle-strand ratio of 5. In the absence of non-conductive obstacles, action potential spread was circular (Fig 2c) and became increasingly anisotropic at higher obstacle-strand ratios, (Fig 2e and 2f), with fastest macroscopic conduction observed along the strands and relatively slower conduction at intermediate angles. Curvature anisotropy of activation isochrones was altered significant at obstacle-to-strand ratios above 3.0 (Fig 2f), with a change from 1.00 (no anisotropy) in the absence of obstacles to 0.85 at an obstacle-to-strand ratio of 7, consistent with diamond-shaped isochrones activation lines (Fig 2e). Spatial distribution of APD was dependent on distance from stimulus site, with largest APD near the stimulus site.

### Mechanisms of conduction slowing

**Fig 3:**
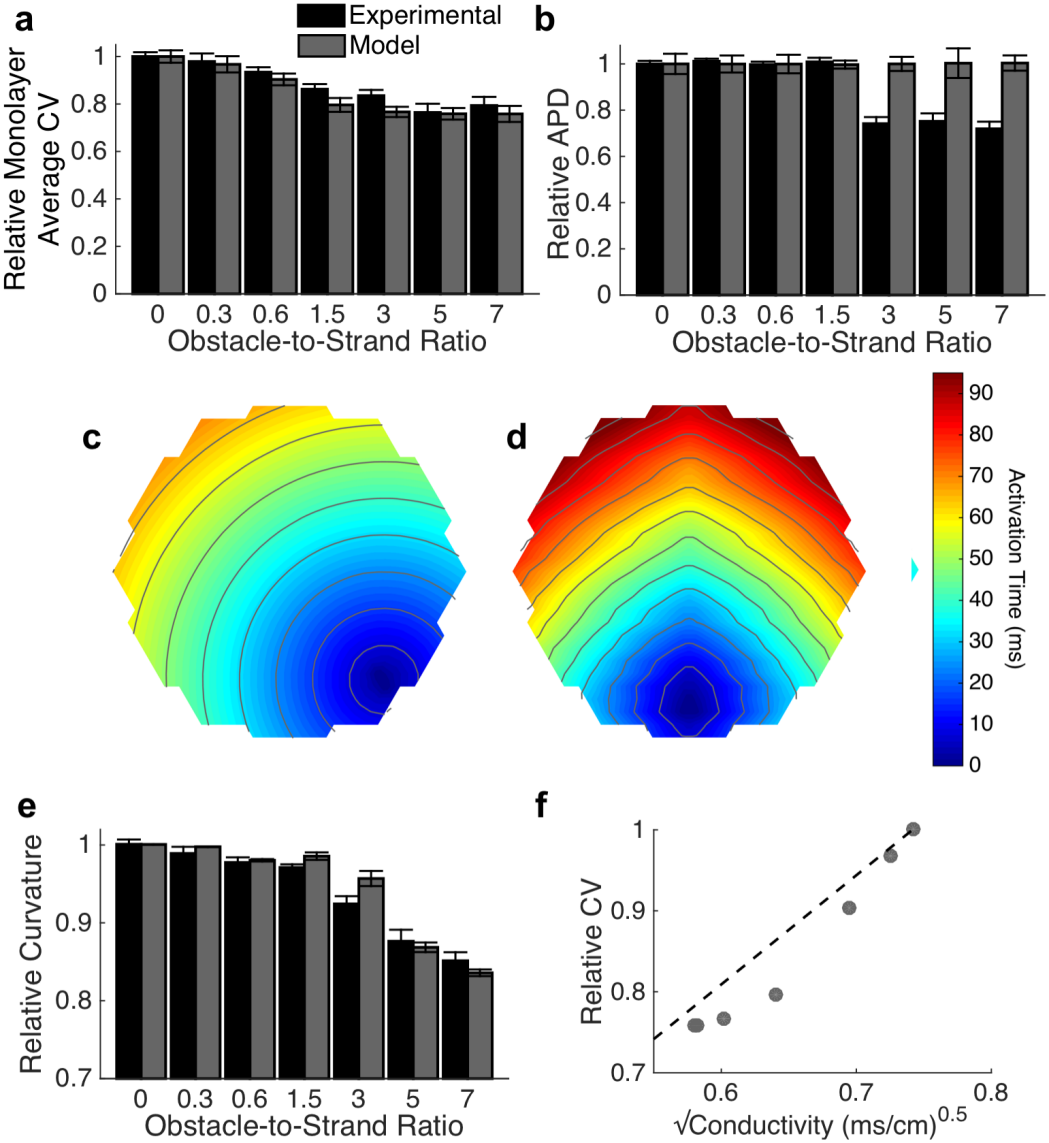
Simulated conduction in microheterogeneous tissues. (a-b) The monodomain biophysical model is able to replicate experimentally observed conduction slowing (a; mean ± se) at increasing obstacle-to-strand ratios, but does not recapitulate the experimentally observed decreased in action potential duration (b; mean ± se). (c-e) Activation isochrones curvatures are qualitatively (c-d for obstacle-to-strand ratios of 0 (c) and 7.0 (d)) and quantitatively (e; mean ± sd) similar to those seen experimentally (Fig 2). Activation isochrone lines at 8 ms spacing (f) Changes in conduction velocity are not completely by the classical square-root relationship between effective electrical conductivity and velocity (R^2^ = 0.86).

Computational models of conduction in microheterogenous Ex293 monolayers were employed to examine mechanisms of conduction slowing observed *in vitro*. A biophysical monodomain model using Hodgkin-Huxley-style ionic behavior (S1 Fig) was applied to the heterogeneous tissue geometry. Multiple simulations were performed for each degree of heterogeneity, with cell-to-cell and monolayer-to-monolayer electrophysiological variation as described in *Methods*, to replicate the variability of experimental observations (n = 10 simulations per case). The biophysical model replicated experimentally observed macroscopic conduction changes due to the presence of microheterogeneities (Fig 3a). However, the model was unable to replicate the 26.1% reduction in APD observed experimentally at the largest obstacle-to-strand ratios (Fig 3b). Activation isochrones (Fig 3c and 3d) and curvature anisotropy (Fig 3e) in simulations qualitatively and quantitatively matched those observed experimentally. To examine whether changes in the macroscopic bulk conductivity can explain conduction slowing, we plotted the relationship between effective electrical conductivity and conduction velocity. Conductivity and velocity failed to follow the classical cable-theory-derived square root relationship; rather, increasing obstacle-to-strand ratio led to larger decreases in velocity than predicted by bulk conductivity (Fig 3f).

**Fig 4:**
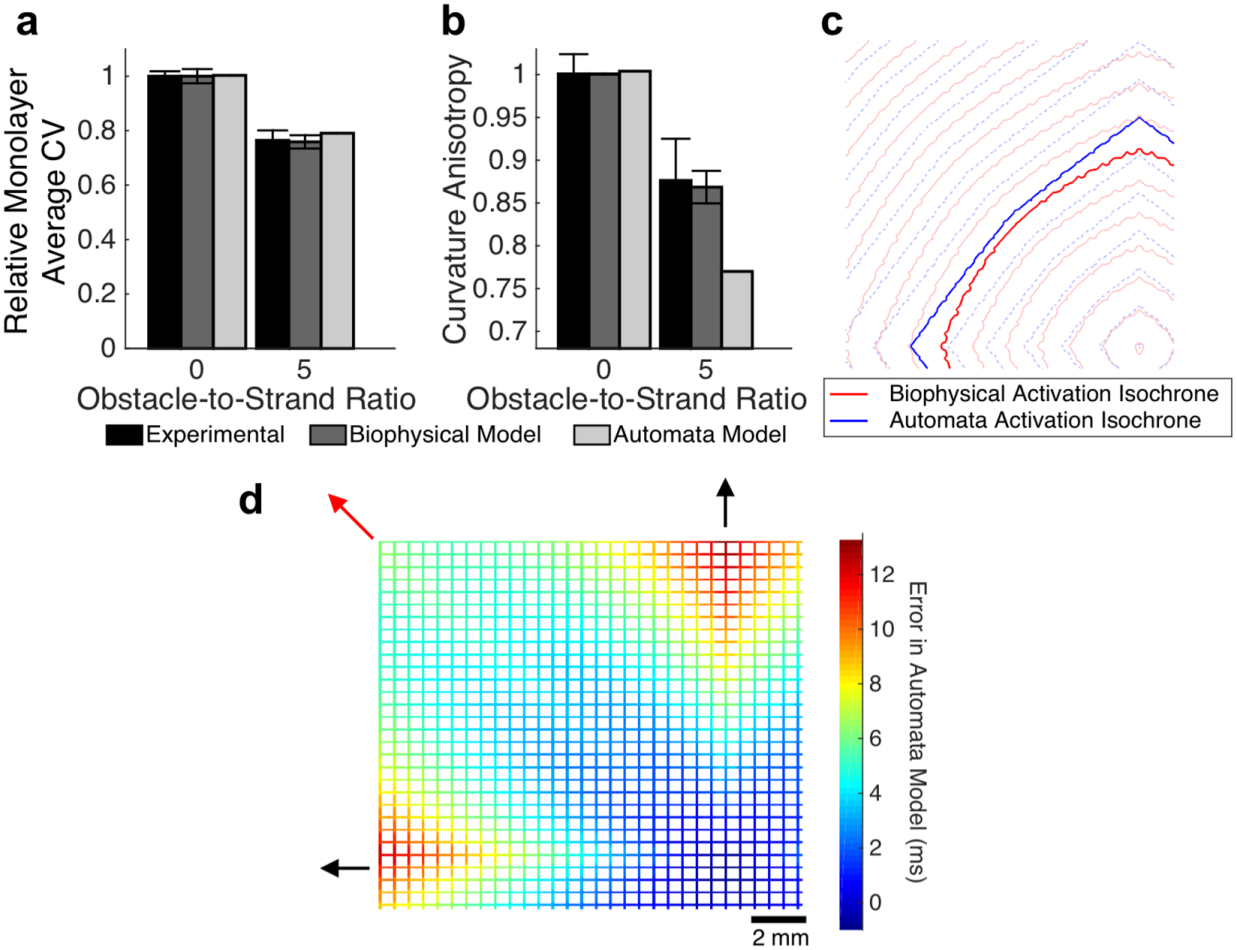
Path tortuosity does not fully explain heterogeneity-induced conduction slowing. (a-b) The automata model, which considers only conduction path length and does not reflect microscopic conduction variation, approximately captures macroscopic conduction velocity (a; mean ± se) but exhibits substantially more curvature anisotropy compared to experimental monolayers and the biophysical model (b; mean ± sd). (c) Activation isochrones are overlaid to highlight the difference in shape between the biophysical model (red) and the automata model (blue). (d). Error in the automata model, compared to the biophysical model, is highest along the principal axes of conductions (black arrows), and lowest along the bisecting diagonal (red arrow), suggesting spatial variation in microscale conduction velocity.

We reasoned that changes in macroscopic conduction velocity with increasing obstacle-to-strand ratio could have originated either from an alteration of conduction path (path tortuosity) or from variation in microscale conduction velocity. To determine whether path tortuosity caused by the presence of non-conductive obstacles is sufficient to explain the slowing seen in microheterogeneous monolayers, a cellular automata model with uniform microscopic velocity was employed. For the case of no obstacles, the calibrated homogenous automata model exhibited a mean absolute activation time error of only 0.3 ms compared to the biophysical model, replicating the isotropic pattern (Fig 4B, left). Even in the presence of acellular heterogeneities with an obstacle-to-strand ratio of 5.0, the automata model exhibited relatively comparable macroscopic conduction slowing relative to homogenous tissue (Fig 4a; 20.9% slowing in automata, compared to 23.6 ± 3.9% slowing in equivalent experimental monolayers and 24.2 ± 3.1% slowing in the biophysical model). However, the automata displayed substantially more curvature anisotropy (Fig 4b; curvature anisotropy ratio of 0.77 compared to mean anisotropy of 0.87 ± 0.05 in experimental preparation and 0.87 ± 0.02 in the biophysical model), suggesting the importance of microscale velocity variation.

**Fig 5:**
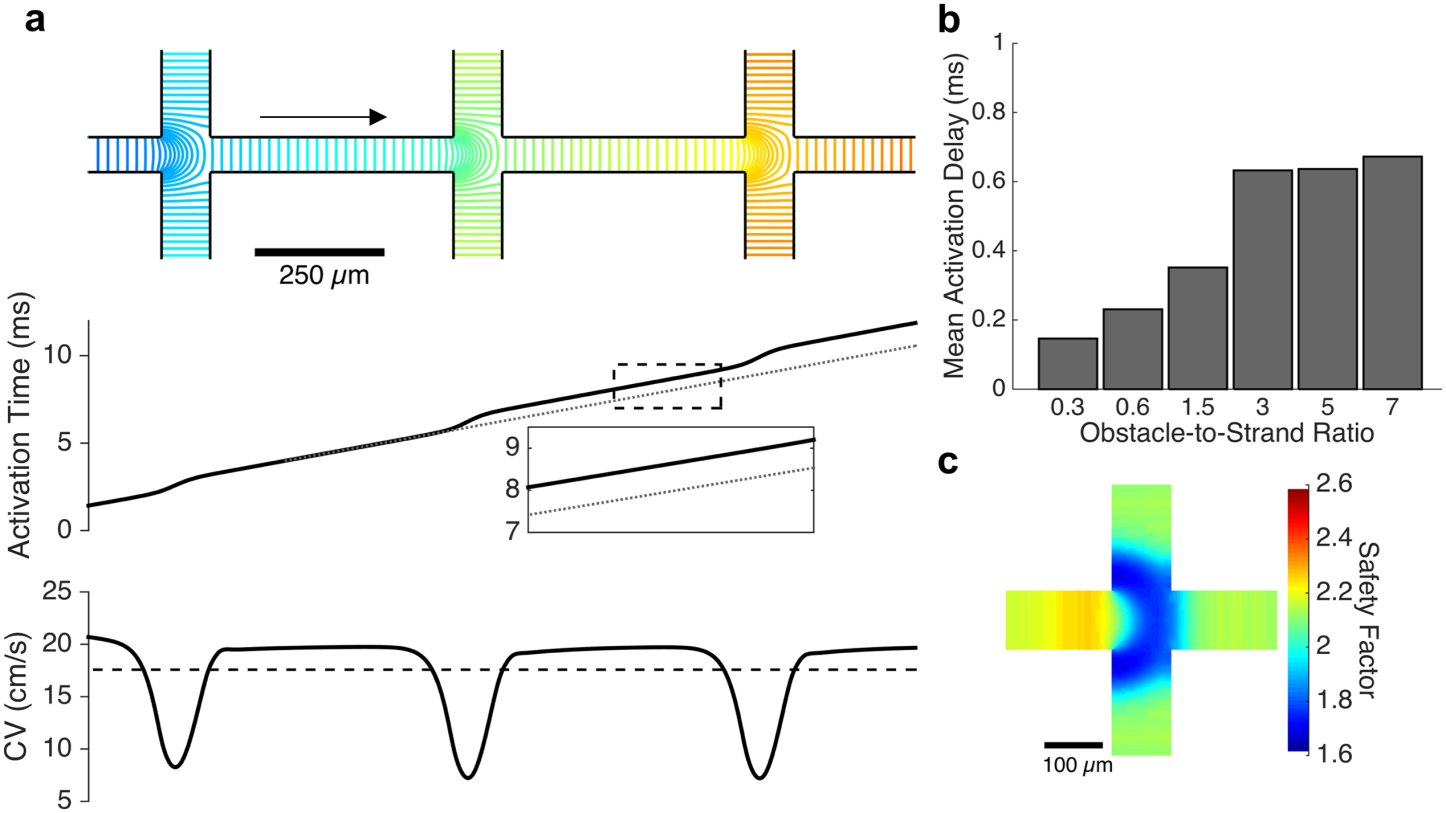
Conduction slowing at branching points. (a) As the wavefront travels from right to left (direction of arrow) along the principal axis of conduction (obstacle-to-strand ratio: 5), activation isochrones reveal significant slowing at each branching point (top and bottom panels). An up to 36.6% decrease in local conduction velocity leads to a conduction delay across the branch point (middle panel) and a net 11.8% reduction in mean CV along the strand (bottom panel, dashed). (b) The magnitude of the conduction delay at each branching point is correlated with the obstacle-to-strand ratio of the tissue. (c). A significant decrease in safety factor of conduction is observed at each branch point, indicating that a mismatch of electrical source and load is responsible for the conduction delay.

Microscale conduction behavior was therefore examined at several key regions of the simulated monolayers. Activation patterns directly along the principal axes (directly in line with the stimulus site) revealed significant conduction slowing at each branching point where the wavefront exits from between two neighboring obstacles (Fig 5a, top). In the tissue with an obstacle-to-strand ratio of 5.0, the local CV decreased from a mean of 19.7 cm/s in the middle third of each intra-obstacle strand to a minimum of 7.20 cm/s at the strand intersection, resulting in an overall mean CV of 17.5 cm/s along the length of the strand (Fig 5a, bottom). Slowing at each branching point led to an activation delay across the intersection; the magnitude of this delay increased with increasing obstacle-to-strand ratio, from 0.15 ms per branching point in tissues with a ratio of 0.3, to 0.63 ms per branching point at a ratio of 3.0 (Fig 5b). Further increases in obstacle-to-strand ratio resulted in minimal increases in activation delay. The safety factor of conduction also sharply decreased at each branching point, with a 21.9% reduction in safety factor for obstacle-to-strand ratios of 3.0 or greater (Fig 5c), though safety factor remained above 1.6 in all cases and did not approach unity, the point of conduction block.

Further examination of electrical properties at a site of branching revealed a decreased upstroke velocity at the entrance to and throughout the branching site (S2d Fig). However, peak sodium current was found to increase in magnitude immediately prior to the branching site before decreasing as the wavefront propagated distally through the intersection (blue arrow in S2f Fig). In addition, action potential duration was found to decrease away from the stimulus site, with step reductions in APD noted at branching sites (S2c Fig). The cumulative conduction delay due to branching along the principal axes is approximately equivalent to the error observed in the automata model (S3 Fig).

**Fig 6:**
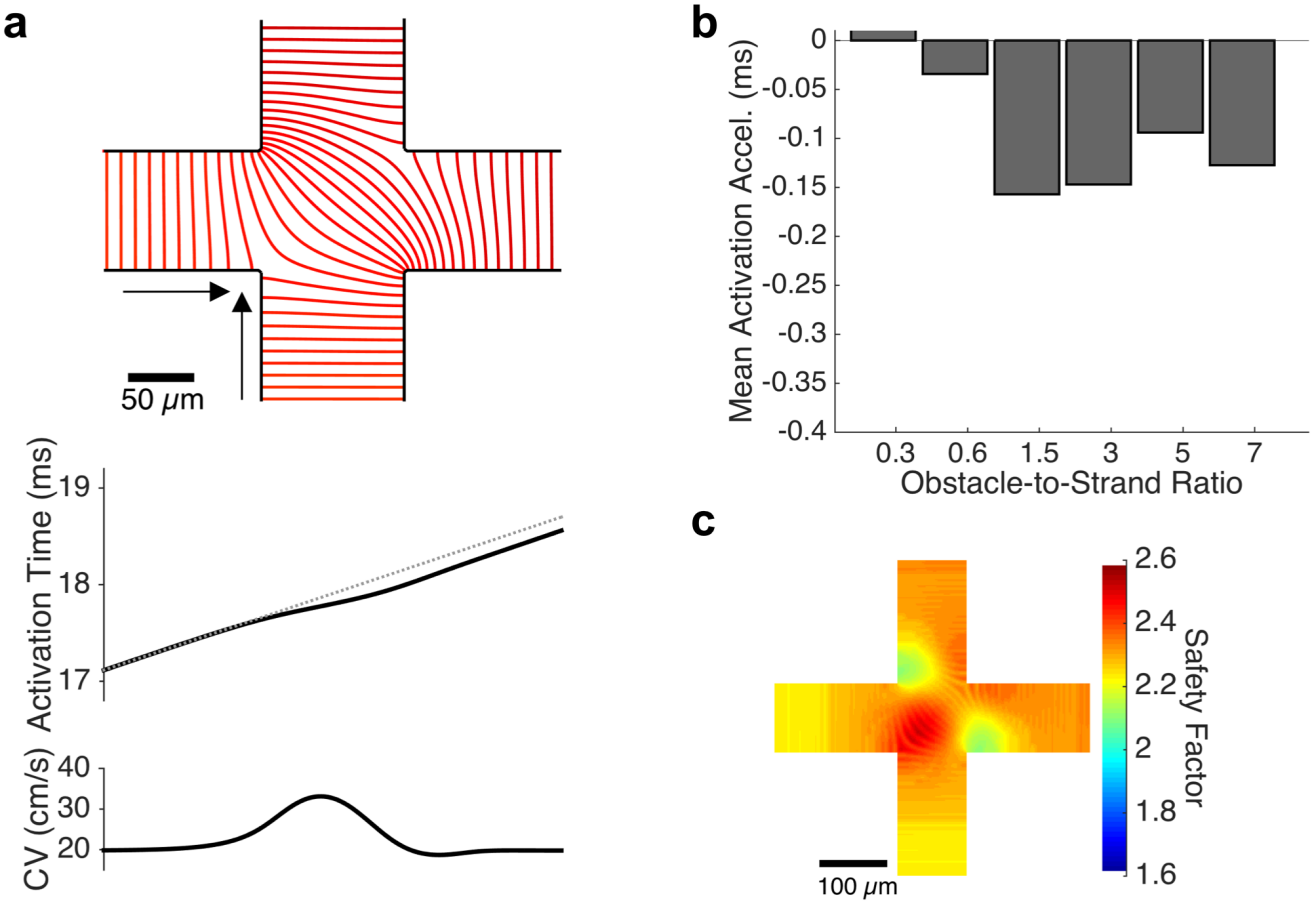
Conduction acceleration due to wavefront collision. (a) At intersection points along the diagonal axes, the simultaneous arrival of two wavefronts leads to a non-annihilating wavefront collision that causes a local increase in conduction velocity. This local speeding, up to 67.3% In tissues with an obstacle-to-strand ratio of 5, leads to an acceleration of activation across the intersection site. (b) The magnitude of acceleration is approximately 0.15 ms for all obstacle-to-strand ratios above 1.5. (c) The safety factor of conduction rises sharply at the site of wavefront collision at the center of the intersection; reduced safety factor is observed near the corners of the intersection.

Away from the principal axes, two wavefronts arrive simultaneously at strand intersection points located along a diagonal from the stimulus site. In the tissue with an obstacle-to-strand ratio of 5.0, the collision of these wavefronts results in a local increase in conduction velocity (Fig 6a) from a mean velocity of 19.8 cm/s within each intra-obstacle strand to a peak of 33.13 cm/s and yields a net acceleration of the wavefront by approximately 0.1 ms per intersection point (Fig 6b). The safety factor for conduction is also increased by the wavefront collision, from 2.27 in the middle third of the approaching strand to a peak of 2.55 within the strand intersection (Fig 6c). Within an intersection point, the location where the arriving wavefronts first collide is associated with rapid local conduction and elevated action potential upstroke velocities (S4 Fig). However, peak sodium current is counterintuitively observed to be smallest in the regions of rapid conduction and largest in magnitude at the areas of reduced conduction velocity (S4f Fig). No significant spatial variation in action potential duration was observed at sites of wavefront collision (S4c Fig). At all strand crossings away from the diagonal and principal strands, the arrival of one wavefront precedes the second wavefront; the difference in arrival time is minimal near the diagonal (where a small degree of wavefront acceleration is observed) and substantial near the primary axis (where wavefront slowing is observed). Thus, microscopic conduction changes at these strand crossing sites in microheterogenous tissue play a significant role in altering macroscopic conduction velocity.

### Regimes of critical conduction: Reduced excitability and coupling

**Fig 7:**
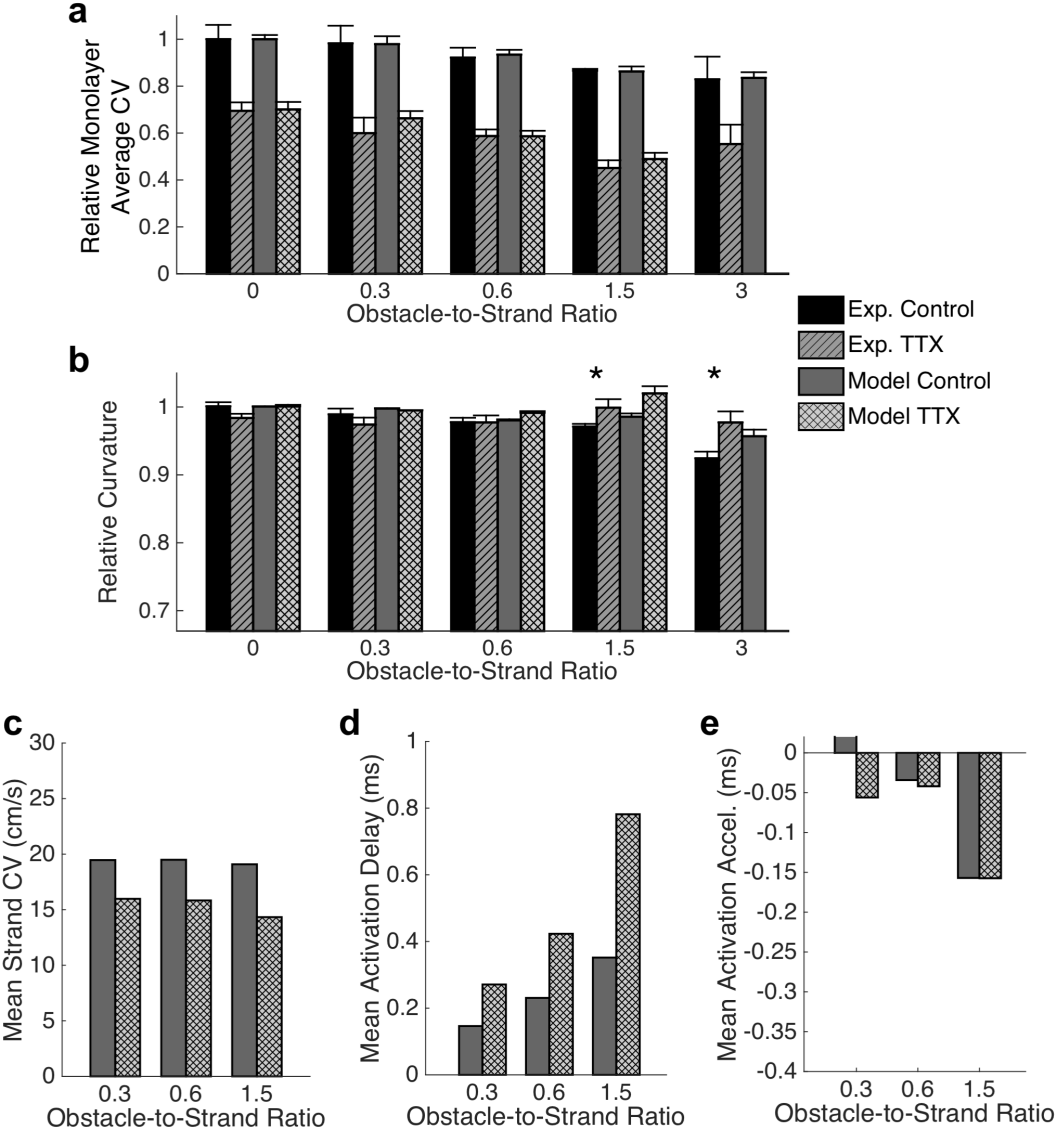
Conduction during reduced excitability. (a) Reduction of excitability via sodium channel blockade with TTX in experimental monolayer results in a significant reduction in conduction velocity (n = 3–6 monolayers; mean ± se; main effect of TTX: F(1,46) = 24.02. p < 0.001; main effect of ratio: F(6,46) = 3.58. p = 0.007; interaction effect: F(6,46) = 0.38. p > 0.1). Conduction could not be reliably sustained at an obstacle-to-strand ratio of 7 and was meandering and irregular with numerous wavebreaks at an obstacle-to-strand ratio of 5. Simulated conduction with reduced excitability replicates experimental behavior at obstacle-to-strand ratios of up to 1.5, but exhibits conduction block at a ratio of 3.0 (n = 10 simulated monolayers per case; mean ± se) (b) Reduced excitability resulted in a reversal of the isochrones flattening observed at large obstacle-to-strand ratios. (n = 3–10 monolayers; mean ± sd; main effect of TTX: F(1,82) = 2.77. p = 0.101; main effect of ratio: F(4,82) = 5.21. p = 0.001; interaction effect: F(4,82) = 4.75. p < 0.002; * indicates significant difference between experimental control and experimental TTX, p < 0.05). Qualitatively similar changes in wavefront curvature are observed in simulations. (c-e). These changes can be attributed to globally reduced strand conduction velocity (c), in conjunction with increased activation delay at branch points along the principal axes (d) and minimal change in collision-induced acceleration along the diagonal (e).

Previous studies have shown that the effects of the microstructure become more critical when excitability is reduced [1]. Reduction in excitability was produced in our experiments by treating the tissue networks with 5 μM tetrodotoxin (TTX) which led to a significant, 33.6%, decrease in conduction velocity (Fig 7a) for obstacle-to-strand ratios from 0 to 5. Conduction could not be sustained at an obstacle-to-strand ratio of 7 in the presence of TTX, and it was already irregular and meandering with numerous localized wavebreaks at an obstacle-to-strand ratio of 5.0. The degree of conduction slowing was not statistically different for obstacle-to-strand ratios between 0 and 3.0. Reduced excitability led to a significant change in the shape of activation isochrones, attenuating the previously observed increase in anisotropy with increasing obstacle-to-strand ratio (Fig 7b), and causing a reversion from diamond-shaped to circular isochrones at the highest ratios (S5 Fig).

We then simulated the experiments with TTX treatment by reducing the conductance of both sodium currents in the Ex293 model. Simulations reproduced experimental effects of reduced excitability on conduction velocity at obstacle-to-strand ratios up to 1.5 (Fig 7a), but resulted in conduction failure at the higher ratios of 3 and 5. When simulated activation times were compared in the presence and absence of TTX at an obstacle-to-strand ratio of 1.5, the activation delay caused by TTX treatment was observed to be anisotropic, replicating the experimentally observed changes in wavefront shape (Fig 7b). Examination of microscopic conduction revealed a 23.8% conduction slowing within intra-obstacle strands (Fig 7c), as well as enhancement of conduction slowing at branch points along the principal axis. In particular, at an obstacle-to-strand ratio of 1.5, microscopic CV in the presence of TTX decreased to as low as 2.6 cm/s resulting in a mean conduction delay of 0.78 ms at each branch point, compared to a mean conduction delay of 0.35 ms in the absence of TTX (Fig 7d; compare to Fig 5b). In contrast, no significant change in the degree of mean conduction acceleration at sites of wavefront collision was noted with TTX application at obstacle-to-strand ratios of 0.6 and 1.5 (Fig 7e; compare to Fig 6b). [While a difference in conduction acceleration is noted at a ratio of 0.3, the wide strands and relatively small obstacles in this monolayer make quantification of wavefront acceleration difficult.] These results suggest that in microheterogeneous tissues, the change in activation wavefront curvature observed with reduced excitability is caused by asymmetric modulation of conduction at branch points and collision sites, resulting in a relatively exaggerated effect of wavefront collisions and a return to rounded isochrones.

We further examined, *in silico*, the effects of reduced coupling on conduction. Reductions in coupling led to non-linear slowing of conduction velocity that was equivalent across obstacle-to-strand ratios (S6a Fig). Minimal change in action potential duration was noted until coupling was reduced by approximately 50% At the largest reductions in coupling, APD was found to prolong, with the effect of reduced coupling largest at the highest obstacle-to-strand ratios. (S6b Fig). The effect of coupling on curvature anisotropy was also examined. At low obstacle-to-strand ratios, the reduction in coupling had minimal impact on curvature anisotropy. However, at intermediate and high obstacle-to-strand ratios, a reduction in couple resulted in a large change in curvature anisotropy (S6c Fig). This change was most profound at the obstacle-to-strand ratio of 3.0 where the curvature anisotropy decreased from 0.96 to 0.82 with a 5-fold reduction in coupling. Microscale examination showed that this behavior was due to a 55% slowing of conduction within strands, coupled with paradoxically reduced delays at branch sites and increased acceleration at collisions (S6d–S6f Fig).

## Discussion

How microfibrosis and other structural microheterogeneities affect tissue conduction remains poorly understood. In this study, we performed a combination of experimental studies in micropatterened cell monolayers and simulation studies in corresponding computational models to examine the effects of acellular heterogeneities on micro- and macroscopic conduction in the setting of normal and reduced excitability. We utilized the excitable Ex293 cell line [28] that replicates key components of the cardiac action potential including a rapid upstroke velocity and relatively fast conduction. Due to their monoclonal origin and expression of only 2 dominant currents, the Ex293 cells have highly homogenous functional properties, which along with an idealized micropatterned structure, allowed us to develop a quantitatively-matched computational model to specifically examine roles of tissue microstructure in action potential propagation.

We found that with an increased ratio of patterned micro-obstacles to surrounding excitable tissue, macroscopic conduction velocity rapidly and then asymptotically decreased while curvature anisotropy gradually increased leading to a switch from circular to diamond-shaped activation isochrones with faster conduction along two principal axes (Fig 2e). This conduction slowing and flattening of isochrone lines was accurately reproduced in biophysical computational models of equivalent tissue geometry. To mechanistically explore these results, we examined whether prolongation of conduction path length around non-conductive obstacles explained the observed changes in conduction by using a cellular automata model [30,31] that effectively removes the effects of source-load electrodynamic interactions. The error between the automata and biophysical models demonstrated that the impact of conduction path distance is insufficient to explain the effects of heterogeneity, and suggested that local micro-scale variations in conduction velocity play a critical role in observed macroscopic behavior.

Microscopic examination of conduction within strands directly emanating from the stimulus site revealed patterns of conduction slowing at each branching point. These branch points are functionally similar to the microscopic “brush” pattern examined by Kucera et al., and rapid conduction slowing approaching the branch point followed by mild conduction speeding entering the distal strand observed in the current study resembled the local “pull-push” behavior they described [20]. Unlike in the Kucera study, all branches in our tissue structure were connected away from the two principal axes; however, the principal axes supported rapid conduction such that when the wavefront approached each branching site from along the axis, the off-branches were at rest and acted as an unexcited load to the approaching wavefront. The cumulative effect of conduction slowing due to repeated branching was shown to be approximately equal to the error in the automata model along the principal axes (S3 Fig), suggesting that microscale slowing at branching sites was the primary mechanism for slowed macroscopic conduction along these axes. As such, the microscale physiology previously identified by others plays a critical role in driving macroscopic conduction behavior.

Action potential duration experiences step-drops at each branching site along these principal axes. APD has been previously shown to decrease moving away from the stimulus site in homogenous monolayers (for example, [32]); however, here, APD appears to remain constant within strands and drops only at the branching site where the wavefront transitions from linear to convex (S2c Fig). This transition of wavefront curvature is also associated with a decrease in safety factor of conduction (Figs 5c and S2e), likely due to source-load mismatch at the branching site as well as an intrinsic decreased safety of convex wavefronts, consistent with previous studies [33,34].

Along the radial line equidistant from the two principal axes (“the diagonal”), two perpendicular wavefronts approached simultaneously and collided and merged at the entry to the intersection site (Fig 6a). The higher safety factor in these regions suggests that the two convergent concave wavefronts overpower increased electrical load of the strands distal to the intersection site, resulting in conduction speeding. A localized increase in safety factor has previously been described at sites of head-on wavefront collisions [34], but is a novel finding in the context of a concave excitation wavefront. Wavefront collisions leading to locally accelerated conduction have been previously described in the context of multiple reentrant spiral waves in homogeneous tissue [35]. Our studies suggest that collision-driven wave acceleration may also play an important role in maintaining conduction in regions of complex tissue fibrosis and heterogeneity. In these regions, wavefront collisions may perpetuate slow tortious conduction that would otherwise fail due to local source-load mismatch. The combination of slowing along the primary axes due to repeated branchings, and speeding due to repeated wavefront collisions led to activation isochrones that are less anisotropic than predicted by the effect of conduction path length alone (Fig 4).

In the branching sites of the principal axis, reduced conduction velocity was expectedly associated with slowed action potential upstroke and decreased peak sodium current [36]. However, in the regions were the wavefront approached the branches, reduced conduction velocity and slowed action potential upstroke were associated with a seemingly incongruous increase in peak sodium current (blue arrow in S2d Fig). Spach and Kootsey postulated that at the initiation of propagation, the sodium current must discharge both local and downstream capacitance, resulting in an increased sodium current despite reduced upstroke velocity [37]. Since the distal branching site represents an increased electrical load and thus increased downstream capacitance, a comparable mechanism is likely in play at sites of tissue branching. The opposite inverse relationship (rapid conduction with reduced peak sodium current) is noted at sites of wavefront collision (red in S4b Fig), analogous to the observations of Spach and Kootsey at sites near annihilating wavefront collisions.

Fibrosis typically occurs in the context of structural heart disease, and along with reduced excitability and altered coupling, is considered as a major contributor to arrhythmogenesis [1]. In our study, increased tissue heterogeneity under condition of reduced excitability yielded gross conduction slowing and higher vulnerability to conduction block that occurred at the largest obstacle-to-strand ratios (Fig 7a). In addition, reduced excitability attenuated the heterogeneity-induced anisotropic flattening of isochrone curvature due to a substantial increase in branch site conduction delay and no change in intersection site conduction acceleration. This differential modulation of conduction may have important implications in the initiation of post-infarction ventricular arrhythmias in the peri-infarct border zone, which is known to exhibit complex microstructure as well as short-term reductions in cell excitability [38–41]. In this context, the preserved acceleration due to wavefront collisions may allow for continued conduction despite the effects of reduced excitability. It is important to note that the specific dimensions of obstacles and strand sizes leading to block under critical conditions of reduced excitability will depend on the dynamics and duration of the action potential [40].

There are several limitations inherent to this study and the model utilized. First, the use of an engineered excitable cell line cultured in an idealized geometry is not a direct correlate of human disease. The structure of the native myocardium is inherently three dimensional, which significantly affects determinations of source-load mismatch and safety factor. In addition, human fibrosis is more complex in nature than the regular pattern of acellular obstacles used here, and consists not only of non-conductive collagen, but also of myofibroblasts that may be coupled with surrounding myocardium, directly affecting cellular dynamics. However, as described above, this platform facilitates mechanistic studies of the effects of microheterogeneities on conduction and may be useful for designing new anti-arrhythmic therapies. The experimental techniques used here could be used in the future to study more complex monolayers with multiple cell types. In addition, the computational techniques would allow for future studies in complex three dimensional tissues models that address many of the limitations of the current study. Second, in experimental preparations, we observed a shortening of action potential duration at the highest obstacle-to-strand ratios that could not be replicated in the computational model. We postulated that this reduction in APD may have resulted from observed changes in cellular alignment (Fig 1b and 1c) at higher obstacle-to-strand ratios. However, an *in silico* representation of cellular alignment showed minimal impact of local conduction anisotropy on APD (S7 Fig). In addition, alignment-induced changes in ion channel expression have previously been shown to prolong rather than shorten APD. While puzzling and warranting further investigation, this discrepancy between the model and experiment is unlikely to alter our primary findings related to changes in wavefront mμ and conduction velocity.

Finally, our model incorporates tissue- and cell- level variation in ionic conductances and tissue-level variation in conductivities of junctions. We chose to include these features because it allows us to explain, capture and reproduce sources of variability in macro-scale experimental recordings of mean CV and APD [29]. These modes of model variation were not included in the high-resolution simulation used for mechanistic analysis, and are not strictly necessary for deriving mechanistic insight. However, future studies examining how small electrophysiological changes affect peak current distribution and safety of conduction in near-failure regimes may be of interest. In addition, in our studies with reduced excitability, experimental monolayers sustained meandering conduction in cases that failed to conduct in computational models. This discrepancy is likely due to the heterogeneous nature of cell-to-cell junctions and cell shape in cultured monolayers. While our model incorporated local variation in ionic conductances, it does not consider cell-to-cell coupling variations or sub-cellular level tissue structure. These features have little impact during normal propagation, but under critically reduced excitability, may lead to local breakthroughs in conduction [26,42]; as such, the use of discrete microstructural models such as [43–45] in future studies may better reproduce experimental findings, at the expense of greater computational load.

In this work, we studied action potential conduction using *in vitro* and *in silico* model tissues with varying degree of heterogeneity. Specifically, we showed that conduction slows and becomes anisotropic with increased presence of non-conducting micro-obstacles, and that microscale branching and non-annihilating wavefront collisions in this setting govern macroscopic changes in propagation. We further showed that differential effects of reduced excitability on branching and collision behaviors results in global conduction slowing and a reversion to isotropic propagation. Studies in this combined experimental and computational platform are expected to advance our understanding of arrhythmogenic substrates in structurally diseased hearts.

## Methods

### Micropatterning of Ex293 monolayers

Microcontact printing of fibronectin was used, as previous described [13,46,47], to generate monolayers with regularly arranged acellular regions of variable size and density. Briefly, the negatives of the desired patterns were fabricated onto silicon wafers in a 10-μm thick layer of photoresist (SU8-10) using standard soft photolithography techniques. Desired patterns included 150 μm x 150 μm obstacles with variably sized separating strands to achieve 0%, 5%, 15% and 35% acellular region density, and larger obstacle regions (300 μm x 300 μm, 500 μm x 500 μm, and 700 μm x 700 μm) with fixed 100 μm strands to achieve higher heterogeneity densities. Patterns were characterized and are identified by their obstacle width to strand width ratio. Poly-dimethylsiloxane (PDMS) stamps were cast against the wafers, coated with a 50 μg/mL fibronectin solution and pressed onto 22 mm diameter PDMS-coated Aclar coverslips to transfer the fibronectic protein pattern. The engineered excitable Ex293 cell lines were derived as previously described [28,48] via stable transfection of Nav1.5, Kir2.1 and Cx43 proteins in HEK293 cells. Ex293 cells were used rather than neonatal cardiac cells because of their simplified ionic mechanisms that facilitate modeling for paired computational-experimental studies, as described in *Discussion*. Ex293 cells were seeded onto the stamped coverslips at a density of 10^5^ cells per cm^2^. On day 3, patterned monolayers were assessed for confluence before start of optical mapping experiments and any monolayers with patterning imperfections were discarded.

### Optical mapping of impulse propagation

Conduction behavior in engineered monolayers was optically mapped with an array of 504 optical fibers (Redshirt Imaging), as previously described [21,28]. Monolayers were incubated in 10 μM Di-4-ANEPPS, a voltage sensitive dye, for 5 minutes before transfer to the recording chamber with perfused 37 °C Tyrode’s solution. Propagation was trigged by stimulus with a bipolar point electrode (10 ms stimulus at 2Hz unless otherwise specified) and fluorescence signals were acquired at a 2.4 kHz sampling frequency. The resulting recordings were analyzed using a custom MATLAB software [21]. Recorded signals were detrended, filtered, and normalized within each optical channel. In some cases, channels with poor signal quality were removed from analysis. Activation time was defined as the time of maximal action potential upstroke velocity and APD_80_ (AP duration at 80% repolarization) was calculated. The mean CV for each monolayer was determined by calculating a velocity vector at each triangular set of three neighboring optical sensors, based on differences in activation times, and determining the mean of the velocity magnitudes across the monolayer [49]. CV and APD_80_ were normalized to the mean values from date-matched homogenous monolayers. Because of the symmetry and idealized nature of the studied geometry, the CV of each monolayer was summarized in a single CV value that facilitated hypothesis testing. To quantify spatial variation in CV, relative curvature anisotropy of activation isochrones was calculated by dividing the radius of the isochrones along a 45° angle by the mean radius along the perpendicular axes of strand orientation, such that a ratio of 1.0 indicates isotropic curvature, while a ratio of 0.707 indicates diamond-like isochrones shape. In addition, directional CVs were calculated in representative cases using activation times from three sensors near the stimulus site and three sensors near the monolayer periphery in the desired direction.

### Construction of equivalent computational models

Computational tissue models were developed using the geometries of the photomasks used for lithography (as described above). Simulated tissues were 2 cm x 2 cm, with a spatial discretization of 10 *μm*. Distinct non-conductive obstacles were added by applying no-flux boundary conditions at the interface of cellular and acellular regions to recreate experimental obstacle configurations. The tissue was assumed to be locally homogenous (continuum model) rather than discrete with individual cells. The Ex293 membrane model with inherent variability, previously described by our group [29], was used to describe membrane excitability. This model includes four membrane currents: two currents conferred via transfected ion channels (I_Na_, a voltage gated sodium current; and I_K1_, an inward rectifying potassium current), and two currents endogenous to the HEK293 cell line (I_Na,wt_, a wild-type voltage gated sodium current, and I_K,wt_, a wild type delayed rectifier potassium current). The baseline model properties were modified via action potential fitting with genetic algorithm search, to reflect an experimentally observed global increase in Ex293 action potential durations. In this model, the mean current densities for each simulated monolayer are selected from a distribution around the baseline model current densities, with a prescribed coefficient of variation (the “inter-monolayer conductance variation”), and the node-to-node conductivity for each monolayer is also similarly varied using (“inter-monolayer conductivity variation”). In addition, cell-to-cell variation of channel conductances is introduced to allow neighboring cells in a single simulated monolayer to have slight variation in their behavior (“cell-cell conductance variation”); no cell-to-cell variation of conductivity within a single monolayer is included. The coefficients of variation of cell-cell conductance variation, inter-monolayer conductance variation, and inter-monolayer conductivity variation were adjusted to 0.125, 0.08 and 0.25, respectively, to match variability observed in experimental control monolayers.

Conduction was simulated in n = 10 monolayers for each obstacle-to-strand ratio. The effective conductivity of the tissue models was measured using the global method of conductivity estimation described by Kim et al [44]. In this method, the intracellular network, stripped of membrane elements, is grounded along one edge and current is injected along the opposite edge; the resulting potential gradient is used to calculate the electrical field strength. The conductivity is then calculated by taking the ratio of the injected current flux (total injected current / cross sectional area) to the electrical field strength.

Microscopic changes in conduction were examined in 0.4 cm x 0.4 cm simulated tissues with a spatial discretization of 2 μm with no variability to allow for visualization of local changes in conduction. Safety factor of conduction was calculated as previously described [33,50]:

$$SF=\frac{\frac{1}{\beta }\int_{A}^{\;}\nabla ∙\sigma_{m}\nabla V_{m}\;dt}{Q_{thr}(t_{A})}$$

Where *A* is the interval of duration *t*_*A*_ from 1% *I*_*m*_ takeoff to zero *I*_*m*_, and *Q*_*thr*_ is the minimum charge required to reach threshold and elicit an action potential for a stimulus of duration *t*_*A*_. *Q*_*thr*_ was calculated over a range of *t*_*A*_ in a model of an isolated single cell.

### Pharmacological studies

Conduction in the context of reduced excitability was examined using drug treatment. 5 μM tetrodotoxin (TTX) (Sigma) was added to the extracellular bath solution, and optical mapping under 2Hz stimulation was performed 5 minutes after drug treatment. To simulate the effect of TTX *in silico*, sodium current density was decreased in the no-obstacle simulation until the conduction velocity matched that measured experimentally in the presence of TTX. This effective reduction in I_Na_ and I_Na, wt_ was then applied to each degree of heterogeneity.

### Numerical methods

All simulations were performed using the Cardiowave software package [51], a numerical simulation system that incorporates numerous modules for various membrane models, time integration methods and linear solvers. Governing equations were discretized using finite differences with no-flux boundary conditions on the domain boundary and around each obstacle. Propagation was simulated using a semi-implicit Crank-Nicholson scheme with adaptive time steps between 100 μs and 2 ms. A biconjugate gradient stabilized method solver with tridiagonal preconditioner was used to simulate each time-step. Potentials were recorded at intervals of 100 μs at selected individual nodes, and also recorded across the domain using spatial averaging by simulated optical sensors, as described previously [29]. Briefly, in order to replicate experimental measurements using optical mapping [14,28], membrane potentials were averaged over circular regions of diameter 1100 μm, arranged in a hexagonal pattern with 750 μm center-to-center spacing. The resulting signals were analyzed using the same software used for experimental recording analysis.

### Automata model

The modified version of an automata model [30,31] with obstacle-to-strand ratios of 0 and 5.0 was constructed using the geometry of the photomasks used for lithography and a spatial discretization of 10 micron. Propagation was simulated by convolution with a 7x7 Gaussian kernel (sigma = 1.7). Each node in the automata model is represented by a single pseudo-voltage integer value that begins at 0 (rest state), rises up to 100 when activated by neighboring nodes (activation), exponentially decays towards rest (repolarization) and exhibits a fixed-duration refractory state. Because the steps of the automata model are not directly related to time, activation “time” for each node in the homogenous automata model was linearly regressed with true activation times from the homogenous biophysical model, and these regression coefficients were used to determine estimated activation times for the automata model with an obstacle-to-strand ratio of 5.0.

### Statistical methods

Results are presented as mean +/- standard error of the mean unless otherwise specified. Evaluation of statistical significance of APD, CV and curvature anisotropy in experimental data was performed using one-way analysis of variance in the absence of pharmacological intervention, and two-way analysis of variance in the presence of TTX, with an alpha value of 0.05 in all cases. When significance was found, a Tukey post-hoc test for multiple comparisons was performed for pairwise comparisons. Evaluation of the linearity between conduction velocity and the root of conductivity was determined by calculation of the R^2^ correlation coefficient.

## Supporting information

S1 Fig Ex293 model properties. (a) Model action potential trace without parameter variation (dashed black) and 80 additional traces with parameter variability (solid). 72/80 of these traces have an action potential duration within 5 ms of the base action potential. (b) Model conductances for the four Ex293 currents that were identified via genetic search algorithm to reproduce the experimentally recorded action potential (top), and selected properties of the resulting simulated action potential (bottom).
(TIF)

S2 Fig Behavior at branching sites. Conduction at branching points (a; obstacle-to-strand ratio: 5.0) leads to slowing (b) and reduced action potential duration (c). This slowing is associated with a decrease in upstroke velocity, from a mean of 174.3 V/s in the middle third of the strand to a minimum of 53.7 V/s (d), as well as a decrease in safety factor of conduction (e). Peak Sodium (I_Na_) current increases from a mid-strand mean of -374.6 μA/cm^2^ to -405.7 μA/cm^2^ as the wavefront reaches the branching site, indicated by the blue arrow (f). Within the branching site, peak sodium current decreases to a minimum of -281.4 μA/cm^2^.
(TIF)

S3 Fig The error in activation times along the principal axis (from Fig 4d) is approximately equivalent to the cumulative delay along the principal axes caused by slowing at branching sites. Arrow indicates direction of propagation.
(TIF)

S4 Fig Behavior at collision sites. During the collision of two simultaneously arriving wavefront (a), regions with high micro-velocity (red in panel b) exhibit rapid action potential upstrokes (d) and elevated safety of conduction (e), but reduced peak sodium current (f). Conversely, regions of conduction slowing where the arriving wavefronts ‘pivot’ around the corners of an obstacle (blue in panel b), are associated with reduced upstroke velocity and reduced safety factor, but increased peak sodium current. Minimally change in action potential duration is observed at sites of collision (c).
(TIF)

S5 Fig TTX alters activation isochrones. Examination of activation isochrones at an obstacle-to-strand ratio of 1.5 without (a) and with (b) 100 μm TTX reveals global conduction slowing and a reversal of heterogeneity-induced curvature anisotropy.
(TIF)

S6 Fig Effect of reduced coupling, *in silico*. (a) Reduced coupling leads to substantial, non-linear macroscopic conduction slowing that is independent of the obstacle-to-strand ratio. A 67.6% mean decease in CV was observed with a 10-fold reduction in coupling. (b) A 10-fold reduction in coupling results in an prolongation of mean action potential duration (APD). APD was prolonged by 5.6% at obstacle-to-strand ratio of zero and by 15.8% at a ratio of 7.0, indicating ratio-dependence of the effect of coupling. (c) Reduced coupling results in minimal change in curvature anisotropy at low obstacle-to-strand ratios, but a substantial change at intermediate and high ratios. The effect is most pronounced at an obstacle-to-strand ratio of 3.0, where the activation isochrones become much more diamond-like with reduced coupling. (d-f) Examination of microscale behaviors in this case (obstacle-to-strand ratio of 3.0; relative coupling of 0.2) reveals globally slowed conduction (d) with paradoxically reduced delays at branching sites (e) and rapid acceleration at collision points (f). Note that alternating obstacle-to-strand ratios are omitted in panels a-c for figure clarity.
(TIF)

S7 Fig Effect of cellular alignment *in silico*. (a) Anisotropy of conductivities (longitudinal / transverse) was defined as a sinusoidal-like function with no anisotropy at intersection sites and peak anisotropy 250 μm from intersection sites. Changing the peak anisotropy of conductivities resulted in minimal change in macroscopic mean APD (b) but did lead to a substantial increase in conduction velocity (c).
(TIF)

S1 Video Propagation in experimental monolayer with obstacle-to-strand ratio of 0. (GIF)

S2 Video Propagation in experimental monolayer with obstacle-to-strand ratio of 1.5. (GIF)

S3 Video Propagation in experimental monolayer with obstacle-to-strand ratio of 7.0. (GIF)
