# Supplementary figures and images for: Microheterogeneity-induced conduction slowing and wavefront collisions govern macroscopic conduction behavior: A computational and experimental study
Source: PLoS Comput Biol. 2018 Jul 16;14(7):e1006276. doi: 10.1371/journal.pcbi.1006276 (PMC6062105; doi:10.1371/journal.pcbi.1006276)

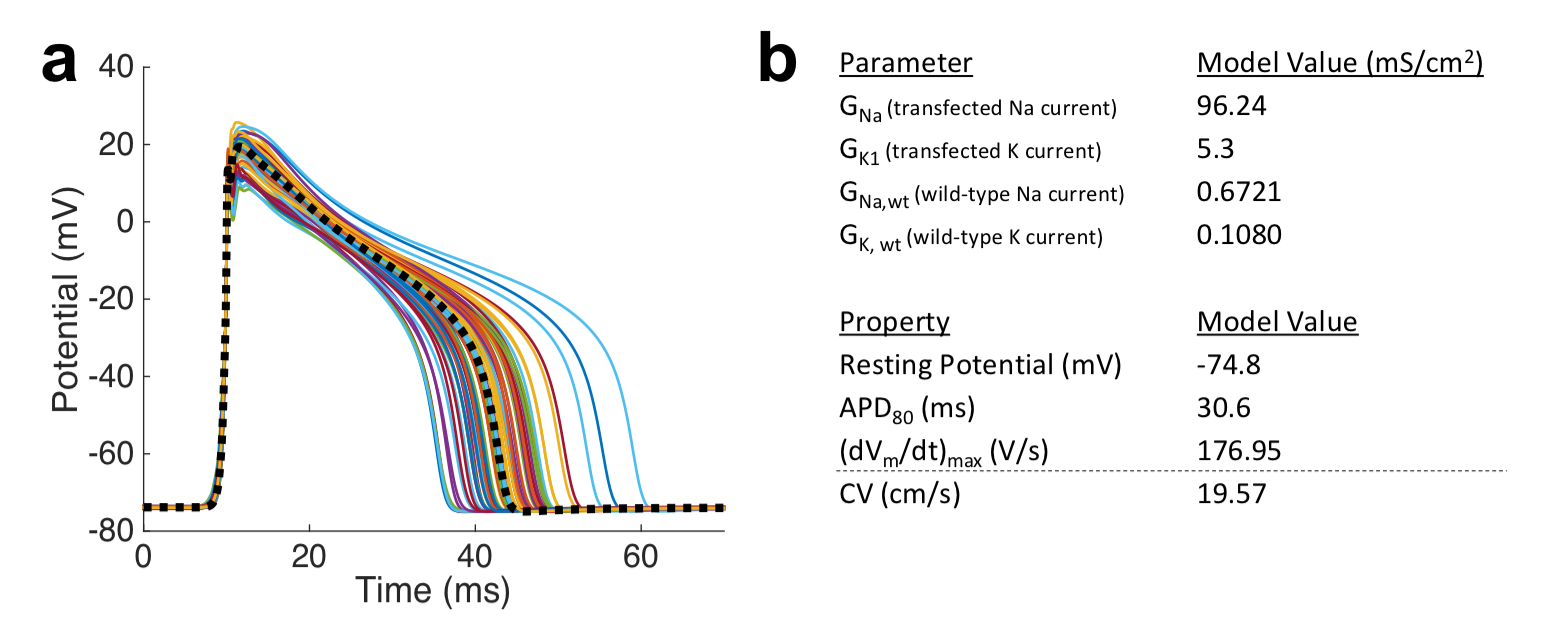

Supplement: S1 Fig — (a) Model action potential trace without parameter variation (dashed black) and 80 additional traces with parameter variability (solid). 72/80 of these traces have an action potential duration within 5 ms of the base action potential. (b) Model conductances for the four Ex293 currents that were identified via genetic search algorithm to reproduce the experimentally recorded action potential (top), and selected properties of the resulting simulated action potential (bottom). (TIF) [file pcbi.1006276.s001.tif]

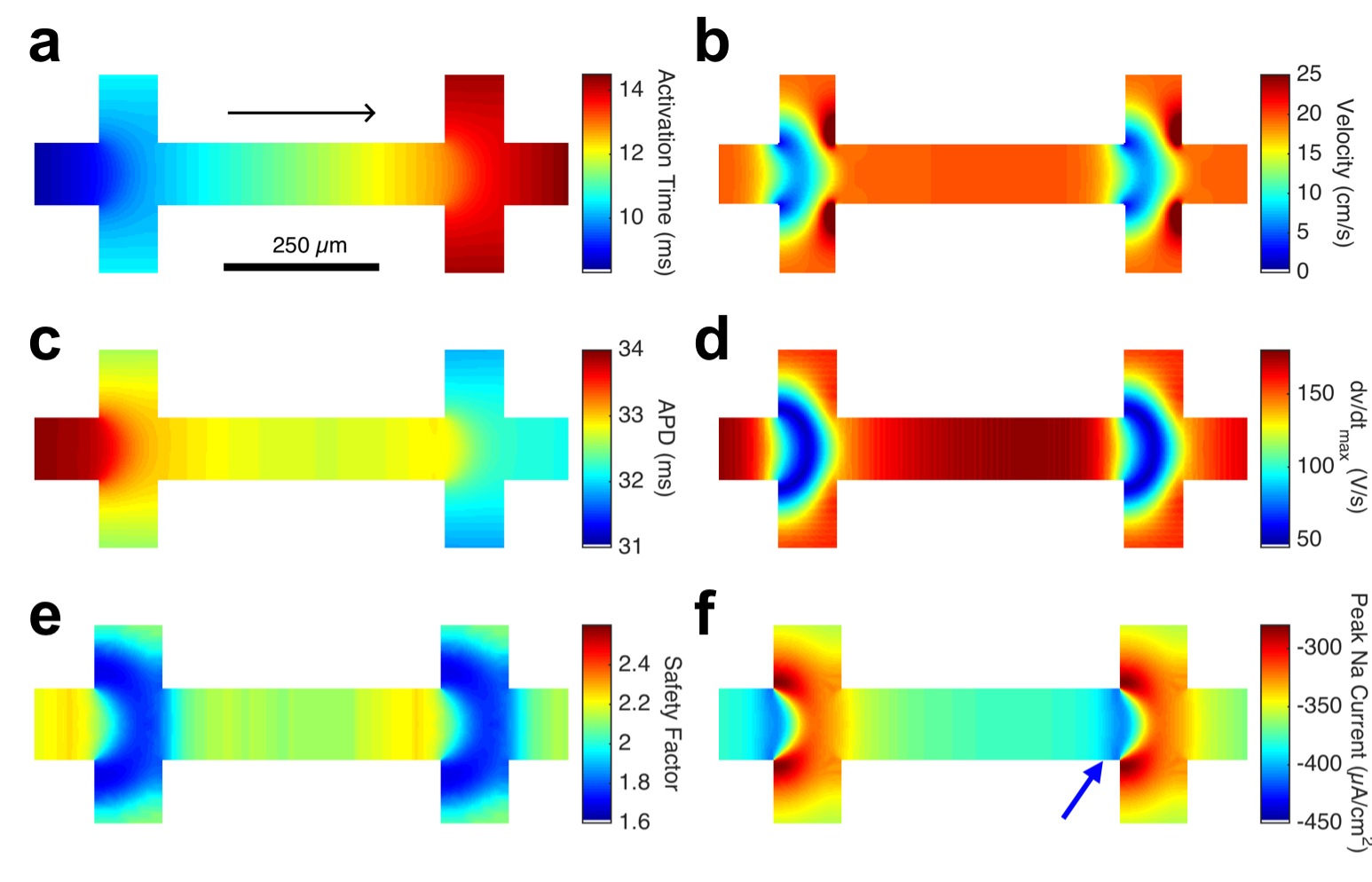

Supplement: S2 Fig — Conduction at branching points (a; obstacle-to-strand ratio: 5.0) leads to slowing (b) and reduced action potential duration (c). This slowing is associated with a decrease in upstroke velocity, from a mean of 174.3 V/s in the middle third of the strand to a minimum of 53.7 V/s (d), as well as a decrease in safety factor of conduction (e). Peak Sodium (INa) current increases from a mid-strand mean of -374.6 μA/cm2 to -405.7 μA/cm2 as the wavefront reaches the branching site, indicated by the blue arrow (f). Within the branching site, peak sodium current decreases to a minimum of -281.4 μA/cm2. (TIF) [file pcbi.1006276.s002.tif]

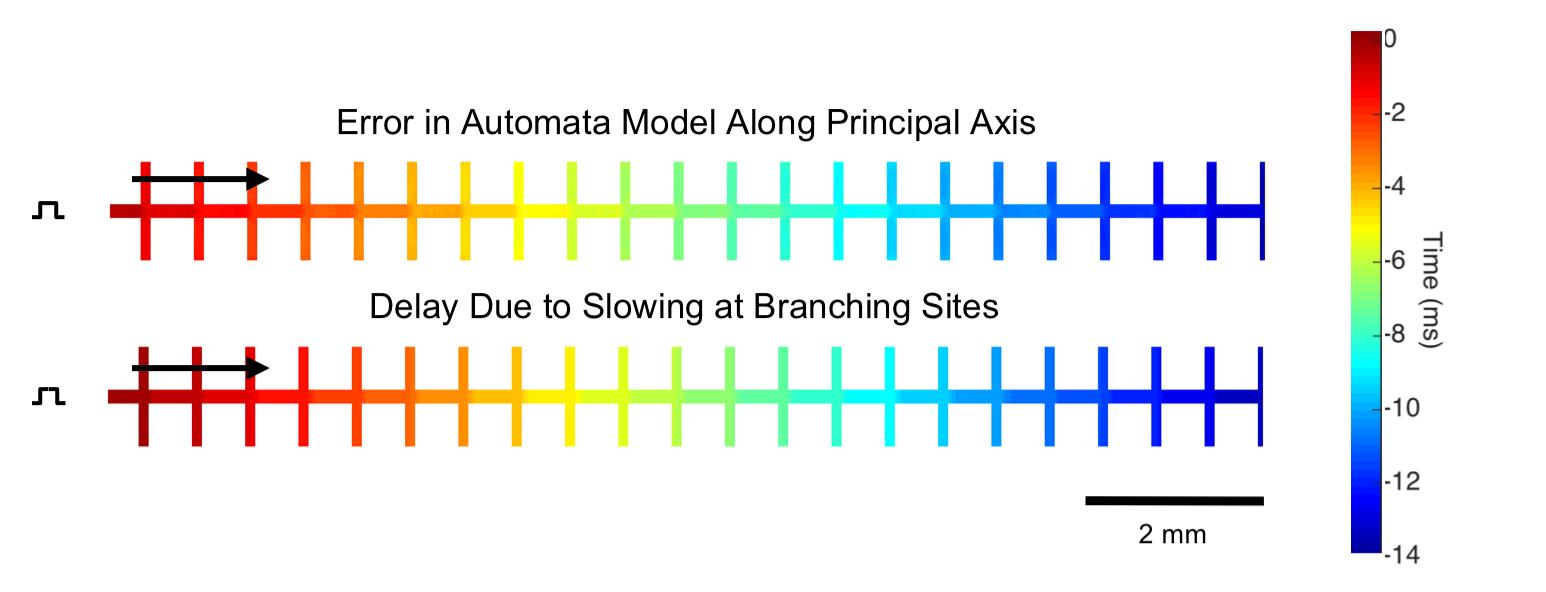

Supplement: S3 Fig — Arrow indicates direction of propagation. (TIF) [file pcbi.1006276.s003.tif]

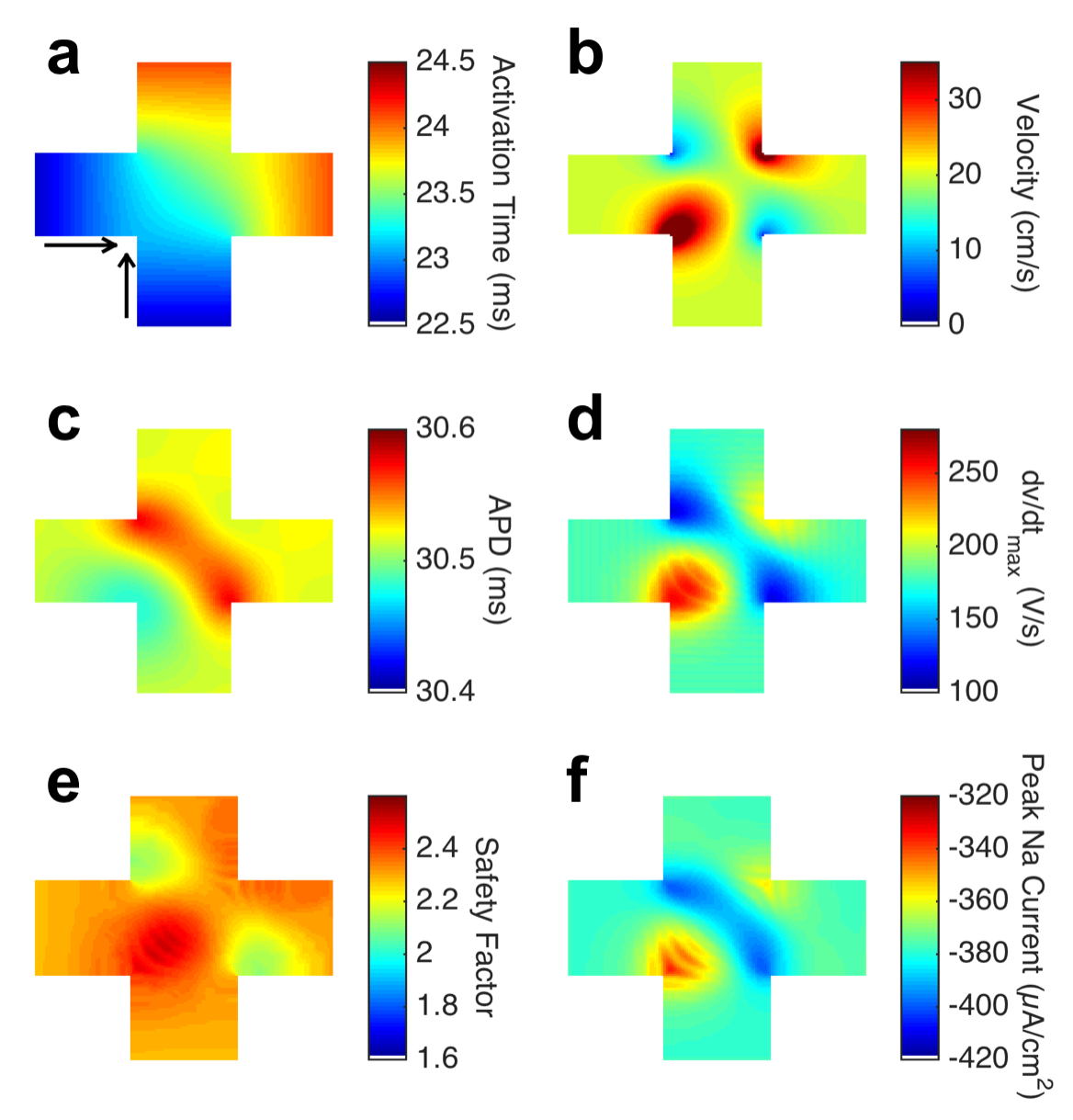

Supplement: S4 Fig — During the collision of two simultaneously arriving wavefront (a), regions with high micro-velocity (red in panel b) exhibit rapid action potential upstrokes (d) and elevated safety of conduction (e), but reduced peak sodium current (f). Conversely, regions of conduction slowing where the arriving wavefronts ‘pivot’ around the corners of an obstacle (blue in panel b), are associated with reduced upstroke velocity and reduced safety factor, but increased peak sodium current. Minimally change in action potential duration is observed at sites of collision (c). (TIF) [file pcbi.1006276.s004.tif]

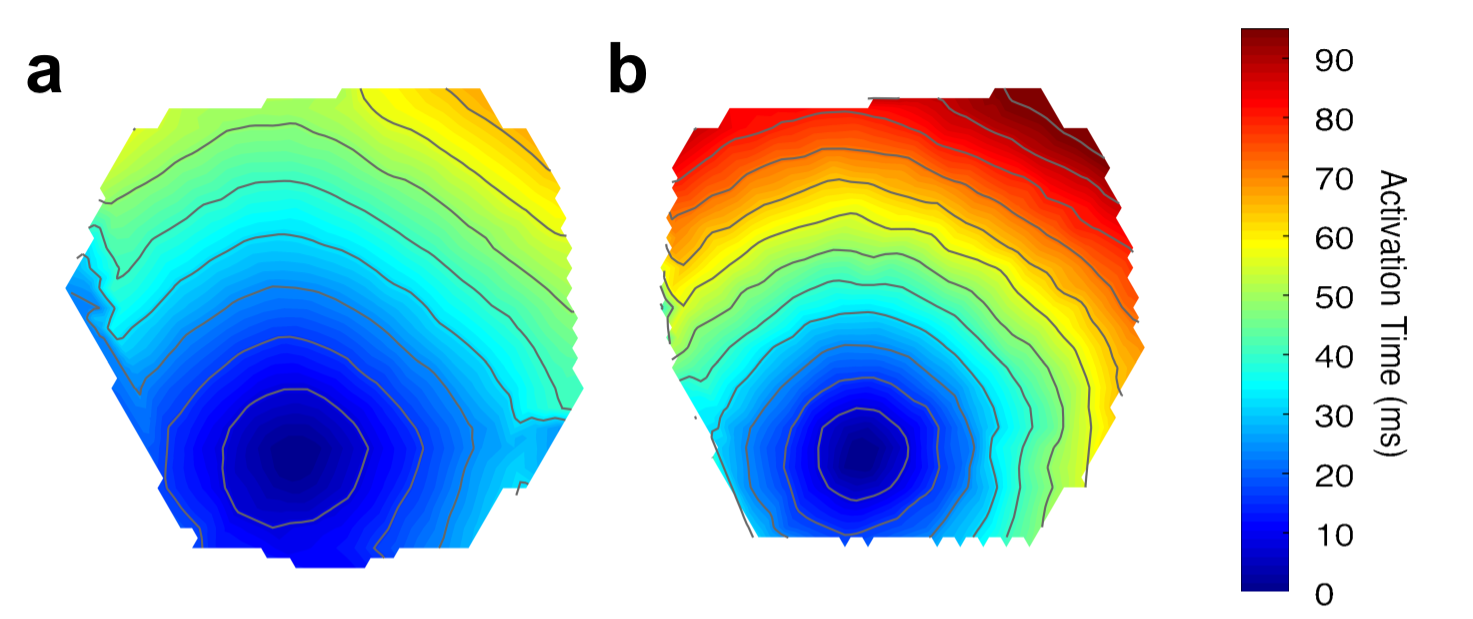

Supplement: S5 Fig — Examination of activation isochrones at an obstacle-to-strand ratio of 1.5 without (a) and with (b) 100 μm TTX reveals global conduction slowing and a reversal of heterogeneity-induced curvature anisotropy. (TIF) [file pcbi.1006276.s005.tif]

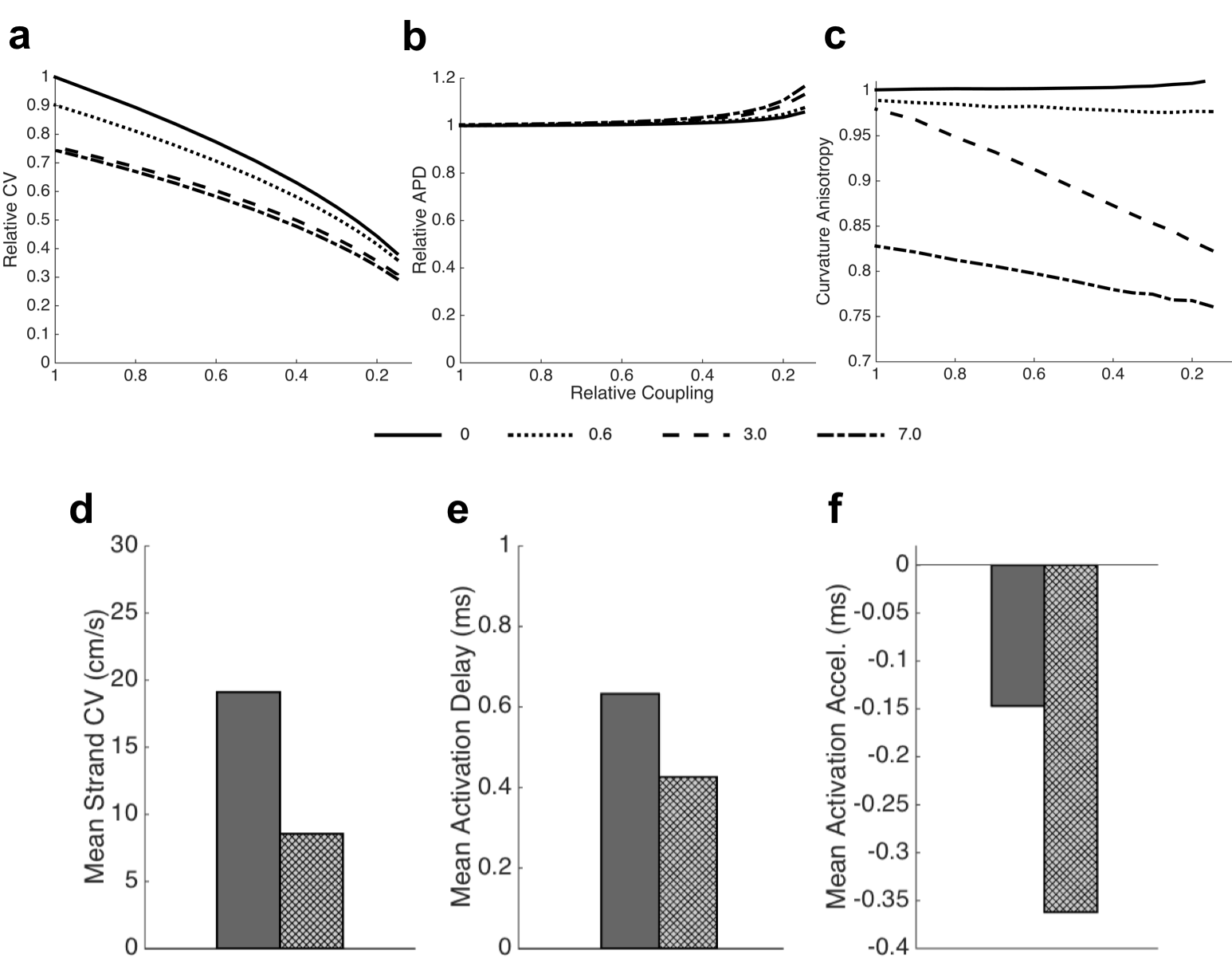

Supplement: S6 Fig — (a) Reduced coupling leads to substantial, non-linear macroscopic conduction slowing that is independent of the obstacle-to-strand ratio. A 67.6% mean decease in CV was observed with a 10-fold reduction in coupling. (b) A 10-fold reduction in coupling results in an prolongation of mean action potential duration (APD). APD was prolonged by 5.6% at obstacle-to-strand ratio of zero and by 15.8% at a ratio of 7.0, indicating ratio-dependence of the effect of coupling. (c) Reduced coupling results in minimal change in curvature anisotropy at low obstacle-to-strand ratios, but a substantial change at intermediate and high ratios. The effect is most pronounced at an obstacle-to-strand ratio of 3.0, where the activation isochrones become much more diamond-like with reduced coupling. (d-f) Examination of microscale behaviors in this case (obstacle-to-strand ratio of 3.0; relative coupling of 0.2) reveals globally slowed conduction (d) with paradoxically reduced delays at branching sites (e) and rapid acceleration at collision points (f). Note that alternating obstacle-to-strand ratios are omitted in panels a-c for figure clarity. (TIF) [file pcbi.1006276.s006.tif]

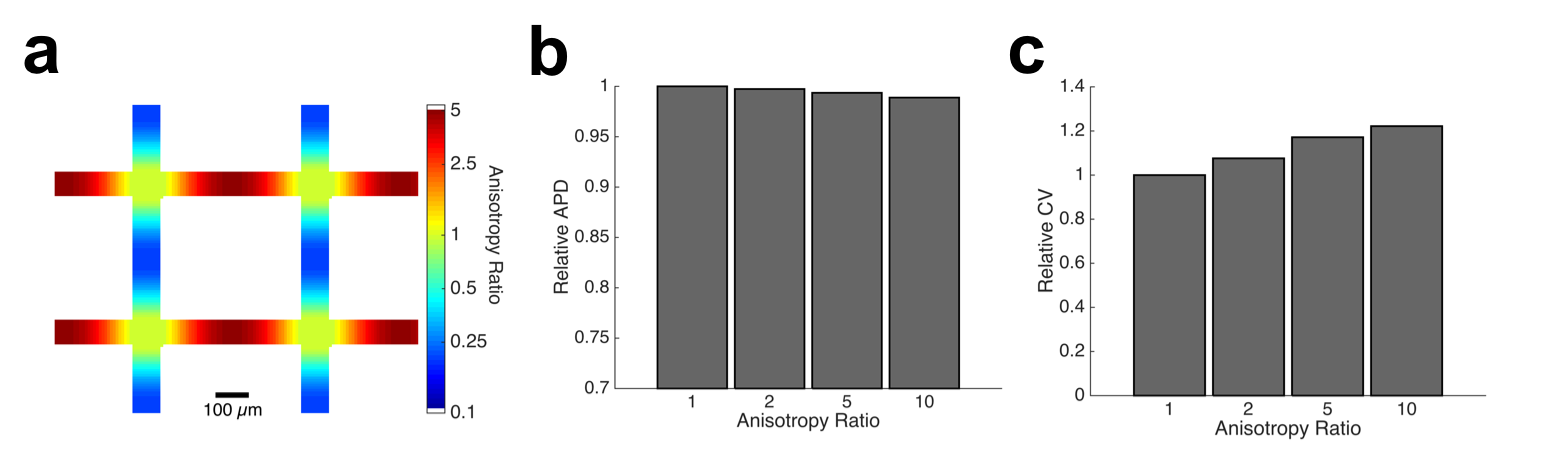

Supplement: S7 Fig — (a) Anisotropy of conductivities (longitudinal / transverse) was defined as a sinusoidal-like function with no anisotropy at intersection sites and peak anisotropy 250 μm from intersection sites. Changing the peak anisotropy of conductivities resulted in minimal change in macroscopic mean APD (b) but did lead to a substantial increase in conduction velocity (c). (TIF) [file pcbi.1006276.s007.tif]

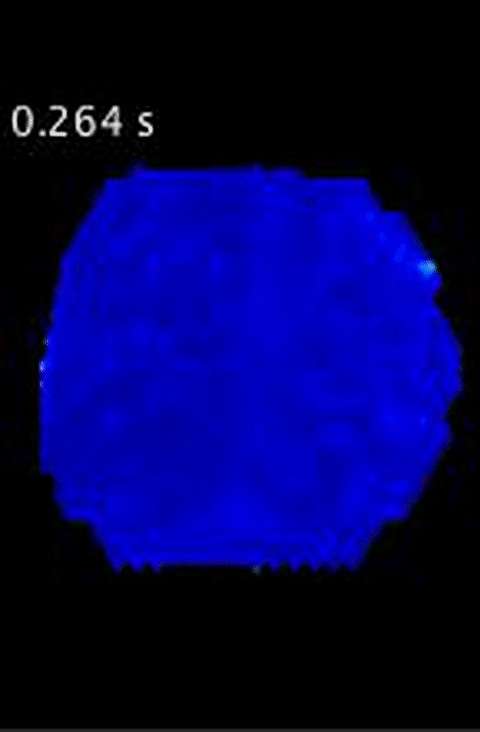

Supplement: S1 Video — (GIF) [file pcbi.1006276.s008.gif]

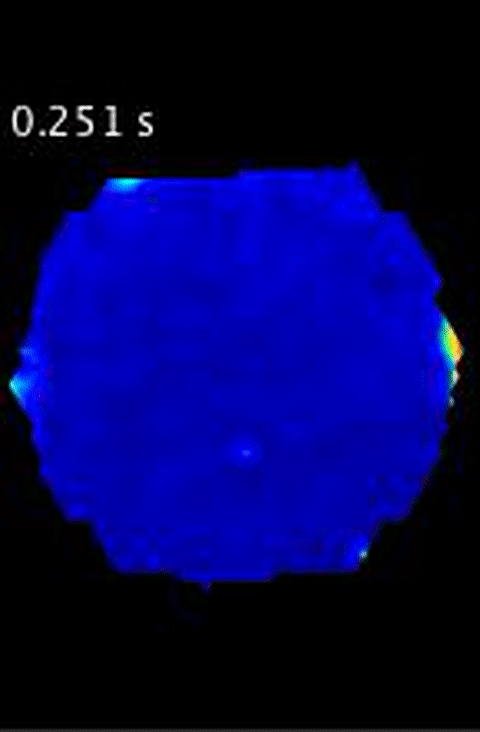

Supplement: S2 Video — (GIF) [file pcbi.1006276.s009.gif]

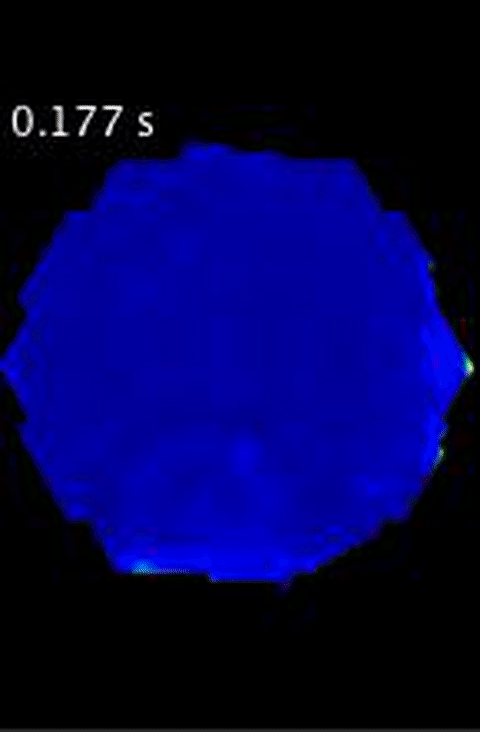

Supplement: S3 Video — (GIF) [file pcbi.1006276.s010.gif]
